# Combination of *in silico* and molecular techniques for discrimination and virulence characterization of marine *Brucella ceti* and *Brucella pinnipedialis*

**DOI:** 10.3389/fmicb.2024.1437408

**Published:** 2024-09-18

**Authors:** Guillaume Girault, Luca Freddi, Maryne Jay, Ludivine Perrot, Alexandre Dremeau, Antoine Drapeau, Sabine Delannoy, Patrick Fach, Acacia Ferreira Vicente, Virginie Mick, Claire Ponsart, Vitomir Djokic

**Affiliations:** ^1^Animal Health Laboratory, EU/WOAH and National Reference Laboratory for Brucellosis, Anses/Paris-Est University, Maisons-Alfort, France; ^2^IdentyPath Genomics Platform, Food Safety Laboratory, ANSES, Maisons-Alfort, France

**Keywords:** *Brucella*, molecular biology, marine mammals, wildlife, molecular typing

## Abstract

**Introduction:**

Mammals are the main hosts for *Brucella* sp., agents of worldwide zoonosis. Marine cetaceans and pinnipeds can be infected by *Brucella ceti* and *B. pinnipedialis*, respectively. Besides classical bacteriological typing, molecular approaches such as MLVA, MLSA, and whole-genome sequencing (WGS) can differentiate these species but are cumbersome to perform.

**Methods:**

We compared the DNA and genome sequences of 12 strains isolated from nine marine mammals, with highly zoonotic *B. melitensis*, *B. abortus*, and *B. suis*, and the publicly available genomes of *B. ceti* and *B. pinnipedialis. In silico* pipelines were used to detect the antimicrobial resistance (AMR), plasmid, and virulence genes (VGs) by screening six open-source and one home-made library.

**Results and discussion:**

Our results show that easier-to-use HRM-PCR, Bruce-ladder, and Suis-ladder can separate marine *Brucella* sp., and the results are fully concordant with other molecular methods, such as WGS. However, the restriction fragment length polymorphism (RFLP) method cannot discriminate between *B. pinnipedialis* and *B. ceti* B1-94-like isolates. MLVA-16 results divided the investigated strains into three clades according to their preferred host, which was confirmed in WGS. *In silico* analysis did not find any AMR and plasmid genes, suggesting antimicrobial susceptibility of marine *Brucella*, while the presence of the VGs *btpA* gene was variable dependent on the clade.

**Conclusion:**

The HRM-PCR and Suis-ladder are quick, easy, and cost-effective methods to identify marine *Brucella* sp. Moreover, *in silico* genome analyses can give useful insights into the genetic virulence and pathogenicity potential of marine *Brucella* strains.

## 1 Introduction

The bacteria of the *Brucella* genus, responsible for a major worldwide zoonosis, can affect farm animals, pets, and wildlife (World Organisation for Animal Health, 2022). Nowadays, the *Brucella* host range is constantly expanding with infection descriptions from aquatic environments and/or from non-mammals (amphibians, fishes, nematodes, trematodes, etc.) (El-Tras et al., 2010; Garner et al., 1997; Hirvelä-Koski et al., 2017; Jaÿ et al., 2020; Lambourn et al., 2013; Mühldorfer et al., 2017; Nymo et al., 2011). To date, 130 species of marine mammals–https://www.fisheries.noaa.gov/, including 86 cetaceans (whales, porpoises, dolphins) and 36 pinnipeds (seals, sea lions, walruses) (Hernández-Mora et al., 2013; Dadar et al., 2023b), are known to be susceptible to *Brucella* infections. Two *Brucella* species are described as having a higher preference for marine ecosystems: *B. ceti* (Cloeckaert et al., 2001), predominantly associated with cetaceans, and *B. pinnipedialis* (Foster et al., 2007), preferentially affecting pinnipeds. Nevertheless, cross-species infections, i.e., *B. ceti* in seals, have also been reported (Maquart et al., 2009), as well as their zoonotic potential (Whatmore et al., 2008). A broad range of pathologies have been associated with *Brucella* infections in cetaceans, with lesions observed from central nervous, respiratory, reticuloendothelial, cardiovascular, musculoskeletal, urinary, and reproductive systems that can cause *Brucella*-induced abortions and considerable fertility decreases (Hernández-Mora et al., 2013). Unlike cetaceans, infected pinnipeds have no clinical symptoms, although suggestive inflammatory lesions have been reported in fur seal aborted pups in Australia (Center for Food Security and Public Health, 2018). In humans, infections with “marine *Brucella* sp. have higher tropism for neural tissues, manifested as severe forms of neuro-brucellosis (two cases from Peru) and spinal osteomyelitis (New Zealand) (Whatmore et al., 2008).

According to *in vitro* investigations (Larsen et al., 2013; Nymo et al., 2016), pathogenicity and zoonotic potential differ based on the host species, e.g., attenuated virulence of *B. pinnipedialis* strains or rather rare naturally acquired *B. ceti* infections in humans (Sohn et al., 2003; McDonald et al., 2006).

Bacteriological evidence for discriminating between *B. ceti* and *B. pinnipedialis* is mainly based on the need for carbon dioxide and the ability to metabolize D-galactose of pinniped strains (adaptation to higher ocean depths), unlike cetacean strains (Banai and Corbel, 2010; Foster et al., 2007; Guzmán-Verri et al., 2012; Jacques et al., 2007; Jahans et al., 1997; World Organisation for Animal Health, 2022).

PCR amplification, based on the fragment size differences, of only 19 bp, is very short to unequivocally differentiate between both marine species (Mayer-Scholl et al., 2010). In addition, the diagnostic tool Bruce-Ladder, recommended by WOAH as a one-step identification test (World Organisation for Animal Health, 2022), is unable to differentiate *B. ceti* from *B. pinnipedialis.*

According to host preferences and ocean distribution (Nymo et al., 2011; Suárez-Esquivel et al., 2017), within each marine *Brucella* species molecular evidence supports the existence of two distinct groups, between cetaceans and pinnipeds, and the probable existence of biovars (Moreno et al., 2002). Multi-locus sequence analyses (MLSAs), multiple-locus variable number of tandem-repeat analysis (MLVA), RFLP (Whatmore et al., 2007; Maquart et al., 2009; Guzmán-Verri et al., 2012; Groussaud et al., 2007; Cloeckaert et al., 2001), and WGS studies (Audic et al., 2011; Wattam et al., 2014) identified five different groups: three clusters among *B. ceti* isolates—*B. ceti* dolphin type [ST26], *B. ceti* porpoise type [ST 23], and *B. ceti* human type [ST 27]; and two clusters among *B. pinnipedialis* isolates—*B. pinnipedialis* hooded seal type [ST 24] and *B. pinnipedialis* common seal type [ST 25]. Besides, phylogenetic investigations underlined that the *B. ceti* dolphin type early diverged, followed by *B. pinnipedialis* and then the *B. ceti* porpoise type, complicating the identification of species-specific markers (Whatmore et al., 2017) and design of one-step species-identification tools, easier and more cost-effective than multiplex assays.

Further genomic comparative analyses showed a genetic proximity of marine *Brucella* isolates with *B. suis* isolates (El-Sayed and Awad, 2018; Whatmore et al., 2006; Le Flèche et al., 2006). An updated approach of Bruce-Ladder for discriminating among *B. canis*, all *B. suis* biovars, and *B. microti* (López-Goñi et al., 2011), designated Suis-Ladder, has proven its worth and identifies correctly two species, *B. ceti* and *B. pinnipedialis*, as well as *B. suis*. This genetic proximity with *B. suis* strains raises the question of a possible application of this *suis/canis*-specific method for the differentiation of marine isolates. More recently, Girault et al. developed an HRM-PCR able to discriminate two groups of *B. ceti* and *B. pinnipedialis* (Girault et al., 2022).

With global warming and shrinking environmental niches, it is observed that the prevalence of marine *Brucella* sp. in cetaceans and pinnipeds increases (Garofolo et al., 2020; Kurmanov et al., 2022; Orsini et al., 2022). To understand pathological factors, that may impact global health, recently *in silico* pan-genome functional analysis on available DNA sequences showed 61 genes matching virulence factors genes within multiple bacterial species (Orsini et al., 2022). Out of those, 31 were included in LPS synthesis, 17 were from the effector delivery system, mainly T4SS, and were 90–100% identical to those identified in *B. melitensis* bv 1 or *B. abortus* or *B. suis*, highly virulent *Brucella* sp. In addition, two genes from type III secretion exporters belonging to the flagella pathway identified in *Bartonella* sp. were also identified in the study of Orsini et al. (2022), implying the zoonotic potential of marine *Brucella* sp. and showing the potency of whole genome analyses, not only in phylogenetic but also functional assay studies.

The present study describes the application of molecular tools such as Bruce-ladder, Suis-Ladder, RFLP, HRM-PCR, MLVA, MLST, and whole-genome approach to discriminate between *B. ceti* and *B. pinnipedialis* and its assessment on field marine isolates. Furthermore, in-depth genomic and functional characterization using the available *in silico* approaches were validated using simple experimental molecular approaches.

## 2 Materials and methods

### 2.1 Bacterial cultivation

This study included 12 isolates from nine marine mammals, including cetaceans [*Stenella coeruleoalba* (*n* = 1), *Tursiops truncatus* (*n* = 2), *Phocoena phocoena* (*n* = 3)] and pinnipeds [*Halichoerus grypus* (*n* = 3)] (Table 1). Additionally, the following reference strains were included: *B. ceti* B1-94 (alias: NCTC12894; BCCN 94–74) and M644/93/1 (alias: B14/94, BBCCN 94–75); *B. pinnipedialis* B2-94 (alias: 94–73); *B. melitensis* (16 M); *B. abortus* (544); and *B. suis* (Thomsen). All strains were cultivated on blood agar base (Thermo Fisher Scientific, France) plates with 5% horse serum and incubated at 37°C under 5% CO2 for 4 days.

**Table 1:** Phenotypical features and details of isolates investigated in this study.

Sample ID	Accession number	Host	Isolation source	Location	Year of isolation	Gram	R/S	CO_2_	H_2_S	Oxidase	Urease	A	M	T20	T10	F20	F10	Tb	Tb 10^4^	Wb	Iz	Species
B1-94 (94–74)	ACEK00000000	*Phocoena phocoena*	NR	NR	NR	cb-	S	−	−	+	+	+	−	+ (+)	+ (+)	+ (+)	+ (+)	−	+w	+w	−	*B. ceti*
M644/93/1 (94–75)	ACBO00000000	*Delphinus delphis*	NR	NR	NR	cb-	S	−	−	+	+	+	−	+ (−)	+ (−)	+ (+)	+ (+)	−	+	+w	+	*B. ceti*
B2-94 (94–73)	*NC_015857.1, NC_015858.1*	*Phoca vitulina*	NR	NR	NR	cb-	S	+	−	+	+	+	−	+ (−)	+ (−)	+ (−)	+ (−)	−	+	+w	+	*B. pinnipedialis*
97–7,763-2	ERS16791920	*Tursiops truncatus*	spleen	France	1997	cb-	S	−	−	+	+	+	−	+ (+)	+ (+)	+ (+)	+ (+)	−	+w	+w	−	*B. ceti*
05–684-1145 R1F	ERS16791921	*Phocoena phocoena*	kidney	France	2005	cb-	S	−	−	+	+	+	−	+ (+)	+ (+)	+ (+)	+ (+)	−	+ w	+ w	+	*B. ceti*
05–684	ERS16791922	*Phocoena phocoena*	liver	France	2005	cb-	S	−	−	+	+	+	−	+ (+)	+ (+)	+ (+)	+ (+)	−	+ w	+ w	+	*B. ceti*
05–684-1143 F1F	ERS16791923	*Phocoena phocoena*	liver	France	2005	cb-	S	−	−	+	+	+	−	+ (+)	+ (+)	+ (+)	+ (+)	−	+ w	+ w	+	*B. ceti*
05–684-1144 F2TM	ERS16791924	*Phocoena phocoena*	liver	France	2005	cb-	S	−	−	+	+	+	−	+ (+)	+ (+)	+ (+)	+ (+)	−	+ w	+ w	+	*B. ceti*
09–601-1272	ERS16791925	*Tursiops truncatus*	spleen	France	2009	cb-	S	−	+	+	+	+	−	+ (−)	+ (−)	+ (−)	+ (−)	−	+ w	−	+ w	*B. ceti*
12-1944-A (4453)	ERS16791926	*Phocoena phocoena*	NR	United Kingdom	2012	cb-	S	+	−													*B. ceti*
12-1944-B (6186)	ERS16791927	*Phocoena phocoena*	NR	United Kingdom	2012	cb-	R	+	−													*B. ceti*
14–901	ERS16791929	*Halichoerus grypus*	NR	Finland	2014	n/a																*B. ceti*
15–1,242-4197	ERS16791930	*Halichoerus grypus*	NR	Finland	2015	cb-	S	+	−	+	+	+	−	+ (−)	+ (−)	+ (−)	+ (−)	−	+	−	n/r	*B. pinnipedialis*
15–1,242-4198	ERS16791931	*Halichoerus grypus*	NR	Finland	2015	cb-	S	+	−	+	+	+	−	+ (−)	+ (−)	+ (−)	+ (−)	−	+	−	+	*B. pinnipedialis*
15–1717-6196	ERS16791932	*Stenella coeruleoalba*	blood	France	2015	cb -	R	+	−	+	+	+	−	+ (−)	+ (−)	+ (−)	+ (+)	−		+ w	+	*B. ceti*

Cb- = coccobacillus gram negative; R = rough, S = smooth; T = Thionine, F = Fuchsin. blue = no growth. n/a = not available – received only DNA. NR = not reported data. *Phocoena phocoena* (harbor porpoise); *Delphinus delphis* (short-beaked dolphin); *Phoca vitulina* (harbor seal); *Tursiops truncatus* (bottlenose dolphin); *Halichoerus grypus* (gray seal); *Stenella coeruleoalba* (striped dolphin).

### 2.2 Phenotypic identification

All 12 isolates were biotyped using standard procedures, based on CO_2_ requirement, H_2_S production, oxidase test, urea hydrolysis, agglutination with monospecific sera, fuchsin and thionine dye sensitivity, and phage typing (World Organisation for Animal Health, 2022).

### 2.3 Molecular analysis

Genomic DNA was extracted from pure bacterial cultures using the High Pure PCR template preparation kit (Roche Diagnostics, France) according to the manufacturer’s instructions. A total of 15 DNAs extracted from marine mammal strains (*B. ceti* and *B. pinnipedialis*) available in the laboratory were used in this study, including that of reference *B. abortus* bv 1,544, *B. melitensis* bv 1 16 M, and *B. suis* bv 2 Thomsen strains (Supplementary Table S1).

Bruce-Ladder (García-Yoldi et al., 2006), Suis-Ladder (López-Goñi et al., 2011), MLVA-16 (Al Dahouk et al., 2007; Le Flèche et al., 2006), and PCR-HRM using previously developed primers for *B. ceti* 1, *B. ceti* 2 clusters, and *B. pinnipedialis* (Girault et al., 2022) assays were performed as previously described.

RFLP-PCR of *omp2a*, *omp2b,* and *omp31* genes was conducted according to the previously published method (Cloeckaert et al., 1995). PCR products of *omp2a* were digested with restriction enzymes *Sty*I and *Nco*I, *omp2b* with *Eco*RI and *Kpn*I, and *omp31* with *Ava*II and *Hae*III. Each RFLP profile was named according to the previously described nomenclature under format X(Y) (Dawson et al., 2008; Cloeckaert et al., 2001). X(Y) represents the combination of the individual restriction patterns of *omp2a* and *omp2b* genes with *omp2a* profiles in parenthesis.

Maximum parsimony clustering analysis was performed on 237 marine *Brucella* MLVA-16 genotypes (Suárez-Esquivel et al., 2017; Maquart et al., 2009; Isidoro-Ayza et al., 2014; Garofolo et al., 2013) available from the public database [hosted by Paris-Saclay University (Orsay, France): http://microbesgenotyping.i2bc.paris-saclay.fr/], as well as marine *Brucella* reference strains and the 12 isolates investigated in this study, using Bionumerics v7.6.2 (BioMérieux, France).

### 2.4 Whole-genome sequencing and bioinformatics

DNA of the 12 isolates was subjected to WGS using an Illumina Nextera XT kit according to the manufacturer’s instructions. Sequencing was performed on the Illumina MiSeq instrument. Additional 24 available *B. ceti*, *B. pinnipedialis* and *B.* sp. genomes as well as 3 referent strains of *B. abortus* (544), *B. melitensis* (16 M) and *B. suis* (Thomsen) in the National Center for Biotechnology Information (NCBI) Genome database (accessed on May 2023) were used in this study for comparison purposes. Moreover, this study included a total of 42 new strains of *B. ceti* and *B. pinnipedialis* that were described by Orsini et al. (2022) as having no genome quality issues (species assignment, high contamination, and/or low level of completeness). The raw reads of these genomes were downloaded from the Sequence Read Archive (SRA) and assembled by our pipeline described below. A total of 78 marine mammal sequences (*B. ceti*, *B. pinnipedialis,* and *Brucella* sp.) were analyzed in this study (Supplementary Table S1). Chimeric genomes of chromosomes 1 and 2 were generated to compare complete and draft genomes (Huang et al., 2012).

Raw *de novo* assembly was performed using Spades 3.11 (Bankevich et al., 2012). An average number of reads for all sequences was characterized as 511,156, and raw reads were mapped on the 16 M chimeric genome (NC_003317.1; NC_003318.1) using the BWA algorithm in BioNumerics 7.6.2 (Applied Maths, BioMérieux). Then, all the sequences were aligned to identify SNPs, which were filtered according to coverage cutoff, inter-SNP distance, and wrong-call bases (unreliable bases, ambiguous bases, gaps) using the wgSNP module in BioNumerics. A minimum set of position filters were applied on the SNP matrix: (i) contiguous SNPs were removed (if found in a 10 bp-window), (ii) with non-informative SNPs, (iii) a required minimum of 20-fold coverage for each SNP, (iv) ambiguous (i.e., non-ACGT bases), and (v) unreliable bases (i.e., Ns) were discarded. The refined SNP matrix was used to generate a maximum parsimony tree using the maximum parsimony algorithm using the wgSNP module in BioNumerics, allowing phylogenetic analyses.

### 2.5 Antimicrobial resistance, plasmid, and virulence gene detection

For targeting the genes and/or regions potentially involved in the AMR, plasmid identification, and virulence (VG), all *B. ceti*, *B. pinnipedialis,* and *Brucella* sp. genomes selected were screened using Abricate version 1.0.1 (https://github.com/tseemann/abricate) with entries from six defined databases AMRFinderPlus (NCBI) (Feldgarden et al., 2019), Comprehensive Antibiotic Resistance Database (CARD) (Jia et al., 2017), ResFinder (Zankari et al., 2012), virulence factor database (VFDB) (Chen et al., 2016), PlasmidFinder (Carattoli et al., 2014,) and MEGARes 2.00 (Doster et al., 2020), as well as additional in-house database (BRUgenes), that includes 10 potential *Brucella* spp. VGs selected in a recent publication on virulence factors of *B. melitensis* (Rabinowitz et al., 2021), not present in the previous six databases.

### 2.6 Confirmation of the presence of eight virulence genes absent in Abricate analysis

To confirm the absence of non-detected VGs in the VFDB and own databases using Abricate, classical PCR was performed. The DNA of 12 *B. pinnipedialis* and *B. ceti* strains described in this study was used to amplify *btpA*, *cgs*, *kdsA*, *pmm*, *wbkA*, *bpe275*, *ure*, and *vceC*, individually (primers described in Table 2), using previously described protocol (Holzapfel et al., 2018). The 2% agar gel was used to separate the amplicons and visualize them. Additionally, *in silico* PCR using BioNumerics was performed to confirm the presence/absence of these genes.

**Table 2:** PCR primer sequences used for the amplification of the *Brucella* virulence-associated genes of interest.

Target gene	Primer designation	Oligonucleotide sequence (5′-3′)	PCR product (bp)	Reference
BtpA	BtpA-F	TCGTTCAGGATCTAGTCGCC	220	Zhang et al. (2022)
	BtpA-R	ATCGGCAATATTCGCGTCTG		
Cgs	Cgs-F	GATCCGGGTGCGAAGTTTAC	237	Zhang et al. (2022)
	Cgs-R	GCCGATGTGATAAAGCTGCA		
KdsA	KdsA-F	CCCGTTCTGACCGATATCCA	226	Zhang et al. (2022)
	KdsA-R	TGGCCAGAACATTCGGATTG		
Pmm	Pmm-F	GCTCCACCGAAACCGATGC	256	Paixão et al. (2009)
	Pmm-R	TCGCTTTTGCCCCATTGG		
WbkA	WbkA-F	TGCCGTCTCTCTACGAAGGT	143	Mancilla et al. (2012)
	WbkA-R	TTCGGCTACGTTCAGAGGAT		
Bpe215	Bpe215-F	TGTCGCGGTCTATGTCTATC	466	Hashemifar et al. (2017)
	Bpe215-R	AATGAGGACGGGCTTGAG		
Ure	Ure-F	GCTTGCCCTTGAATTCCTTTGTGG	2,212	Hashemifar et al. (2017)
	Ure-R	ATCTGCGAATTTGCCGGACTCTAT		
VceC	VceC-F	CGCAAGCTGGTTCTGATC	482	Hashemifar et al. (2017)
	VceC-R	TGTGACGGGTAATTTGAAGC		

## 3 Results

### 3.1 Newly identified *Brucella ceti* and *Brucella pinnipedialis* strains show phenotypic traits characteristic of their respective species

To ensure adequate species identification, the EU referent laboratory for animal brucellosis strain collection, which includes marine field strains from different geographic origins and various terrestrial animal species, was examined. Their phenotypic features (Table 1) were consistent with previously described marine *Brucella* patterns (Guzmán-Verri et al., 2012; Whatmore et al., 2017). As expected, none of the investigated isolates from marine mammals, except for 09–601-1272, isolated from a common bottlenose dolphin (*Tursiops truncates*), produced hydrogen sulfide, but all made urease. All *B. pinnipedialis* isolates required CO_2_ for growth. Standard phenotypic identification confirmed *B. ceti* or *B. pinnipedialis* species profiles (Table 1).

### 3.2 The Suis-ladder, Bruce-ladder, HRM, and RFLP PCRs allowed the exact identification of marine *Brucella* species

**Figure 1:**
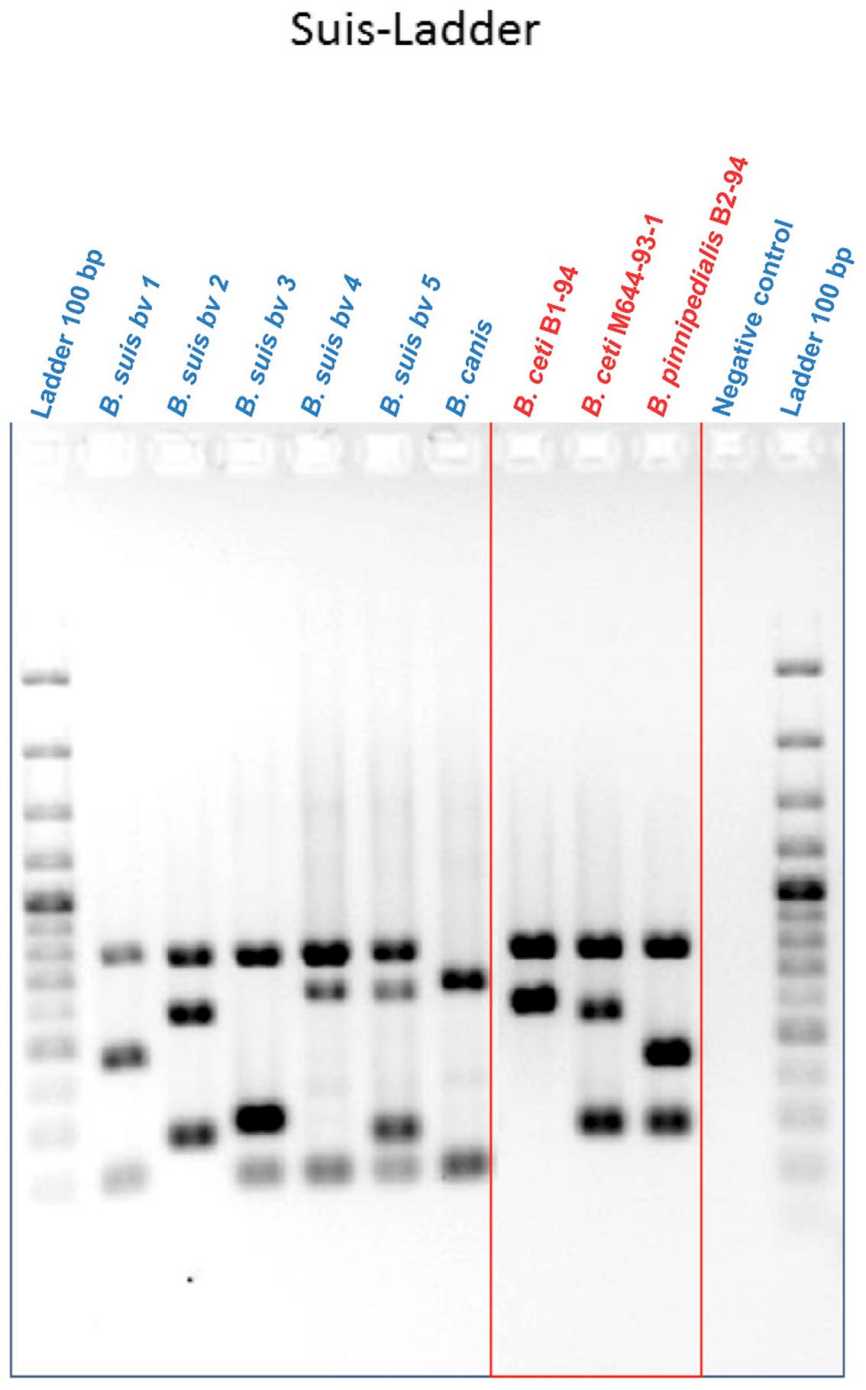
Suis-Ladder patterns of *B. suis* biovars, *B. canis,* and marine *Brucella* reference strains. Lines 1 and 12 contain a 100 bp Ladder. Line 11 contains the negative control, lines 2 to 6 contain DNA from *B. suis* biovar 1, biovar 2, biovar 3, biovar 4, and biovar 5 respectively, line 7 contains DNA from *B. canis*, and lines 8 to 10 contain DNA from *B. ceti* B1-94, *B. ceti* M644-93-1, and *B. pinnipedialis* B2-94, respectively.

The multiplex Suis-ladder PCR, widely used to discriminate among *B. suis* biovars, *B. canis*, and *B. microti*, was performed in order to characterize the marine *Brucella* reference strains *B. ceti* B1-94 (94–74) and M644-93-1 (94–75) and *B. pinnipedialis* B2-94 (94–73) (Figure 1). The *B. ceti* B1-94 (94–74) pattern comprised only two fragments of 774 bp and 550 bp, although the *B. ceti* M644-93-1 (94–75) pattern showed three fragments of 774 bp, 551 bp, and 299 bp. Interestingly, the *B. ceti* M644-93-1 (94–75) pattern shared two fragments with the *B. pinnipedialis* pattern that comprised three fragments of 774 bp, 425 bp, and 299 bp. To ensure adequate species identification using the Suis-Ladder, the ANSES collection of marine field strains from different geographic origins and different host species was examined. Suis-ladder patterns were consistent with the species assigned by biochemical and molecular characterization, and the multiplex assay was able to segregate *B. ceti* isolates into two distinct clusters (Table 3). At the same time, using the Bruce-Ladder, all investigated isolates were confirmed as marine *Brucella,* sharing identical patterns as marine *Brucella* reference strains (Table 3).

**Table 3:** Molecular characterization of marine *Brucella* isolates investigated in this study.

Sample ID	Host	Location	Biotype	Bruce-Ladder	RFLP *omp* pattern	MLVA	WGS	Suis-Ladder	HRM	Concordance
B1-94 (94–74)	*Phocoena phocoena*	NR	*B. ceti*	Marine	M(J)	B	*B. ceti* 1	C1	ceti 1	Yes
M644/93/1 (94–75)	*Delphinus delphis*	NR	*B. ceti*	Marine	N(K)	A	*B. ceti* 2	C2	ceti 2	Yes
B2-94 (94–73)	*Phoca vitulina*	NR	*B. pinnipedialis*	Marine	L(I)	C	*B. pinnipedialis*	P1	Pinni	Yes
97–7,763-2	*Tursiops truncatus*	France	*B. ceti*	Marine	LMOP(IJ)	B	*B. ceti* 1	C1	ceti 1	Yes
05–684-1145 R1F	*Phocoena phocoena*	France	*B. ceti*	Marine	LMOP(IJ)	B	*B. ceti* 1	C1	ceti 1	Yes
05–684	*Phocoena phocoena*	France	*B. ceti*	Marine	LMOP(IJ)	B	*B. ceti* 1	C1	ceti 1	Yes
05–684-1143 F1F	*Phocoena phocoena*	France	*B. ceti*	Marine	LMOP(IJ)	B	*B. ceti* 1	C1	ceti 1	Yes
05–684-1144 F2TM	*Phocoena phocoena*	France	*B. ceti*	Marine	LMOP(IJ)	B	*B. ceti* 1	C1	ceti 1	Yes
09–601-1272	*Tursiops truncatus*	France	*B. ceti*	Marine	N(K)	A	*B. ceti* 2	C2	ceti 2	Yes
12-1944-A (4453)	*Phocoena phocoena*	United Kingdom	*B. ceti*	Marine	LMOP(IJ)	B	*B. ceti* 1	C1	ceti 1	Yes
12-1944-B (6186)	*Phocoena phocoena*	United Kingdom	*B. ceti*	Marine	LMOP(IJ)	B	*B. ceti* 1	C1	ceti 1	Yes
14–901	*Halichoerus grypus*	Finland	*B. ceti*	Marine	LMOP(IJ)	B	*B. ceti* 1	C1	ceti 1	Yes
15–1,242-4197	*Halichoerus grypus*	Finland	*B. pinnipedialis*	Marine	LMOP(IJ)	C	*B. pinnipedialis*	P1	Pinni	Yes
15–1,242-4198	*Halichoerus grypus*	Finland	*B. pinnipedialis*	Marine	LMOP(IJ)	C	*B. pinnipedialis*	P1	Pinni	Yes
15–1717-6196	*Stenella coeruleoalba*	France	*B. ceti*	Marine	N(K)	A	*B. ceti* 2	C2	ceti 2	Yes

NR, not reported data.

In RFLP analysis, only two endonucleases were independently used for each targeted gene in this study (*Sty*I and *Nco*I for *omp2a Eco*RI and *Kpn*I for *omp2b* and *Ava*II and *Hae*III for *omp31*), and results (Table 3) were identified according to the previously described nomenclature (Cloeckaert et al., 2001). Our results showed that *omp31* patterns were strictly identical among *B. ceti* B1-94, *B. ceti* M644-93-1, and *B. pinnipedialis* B2-94, as well as among all investigated field isolates, confirming the absence of polymorphism within *omp31* gene between marine species. Regarding *omp2a* and *omp2b*, only one enzyme per target, respectively *Nco*I and *Kpn*I, was able to discriminate isolates into 2 groups: 3 isolates into N(K) group, including M644-93-1, and 13 isolates, including *B. ceti* B1-94 and *B. pinnipedialis* B2-94, into LMOP(IJ) patterns (Table 3). Nevertheless, the RFLP method applied here did not allow discrimination between *B. pinnipedialis* and *B. ceti* B1-94-like isolates.

Previously published HRM-PCR SNP (Girault et al., 2022) identified all analyzed strains as Marine *Brucella* sp. Furthermore, using specific primers for *B. ceti* 1, *B. ceti* 2, and *B. pinnipedialis*, strains were separated into ST 23, ST 26 – Europe, and ST 24/25 clades, respectively (Table 3). Using B1-94 (94–74) as a reference, strains 97–7,763-2 (dolphin), 05–684-1145 R1F (porpoise), 05–684 (porpoise), 05–684-1143 F1F (porpoise), 05–684-1144 F2TM (porpoise), 12-1944-A (4453) (porpoise), 12-1944-B (6186) (porpoise), and 14–901 (gray seal) were all identified as *B. ceti* 1 (Table 3). Strains 09–601-1272 (dolphin) and 15–1717-6196 (dolphin) were identified as *B. ceti* 2 using M644/93/1 (94–75) as a reference strain (Table 3). At the same time, two strains, 15–1,242-4197 and 15–1,242-4198 from gray seals in Finland, were classified as *B. pinnipedialis* using B2-94 (94–73) as a reference strain (Table 3).

### 3.3 The phylogenetic analyses using WGS classify all publicly available marine strains into three distinct clades, corresponding to *Brucella* populations identified by MLVA-16

**Figure 2:**
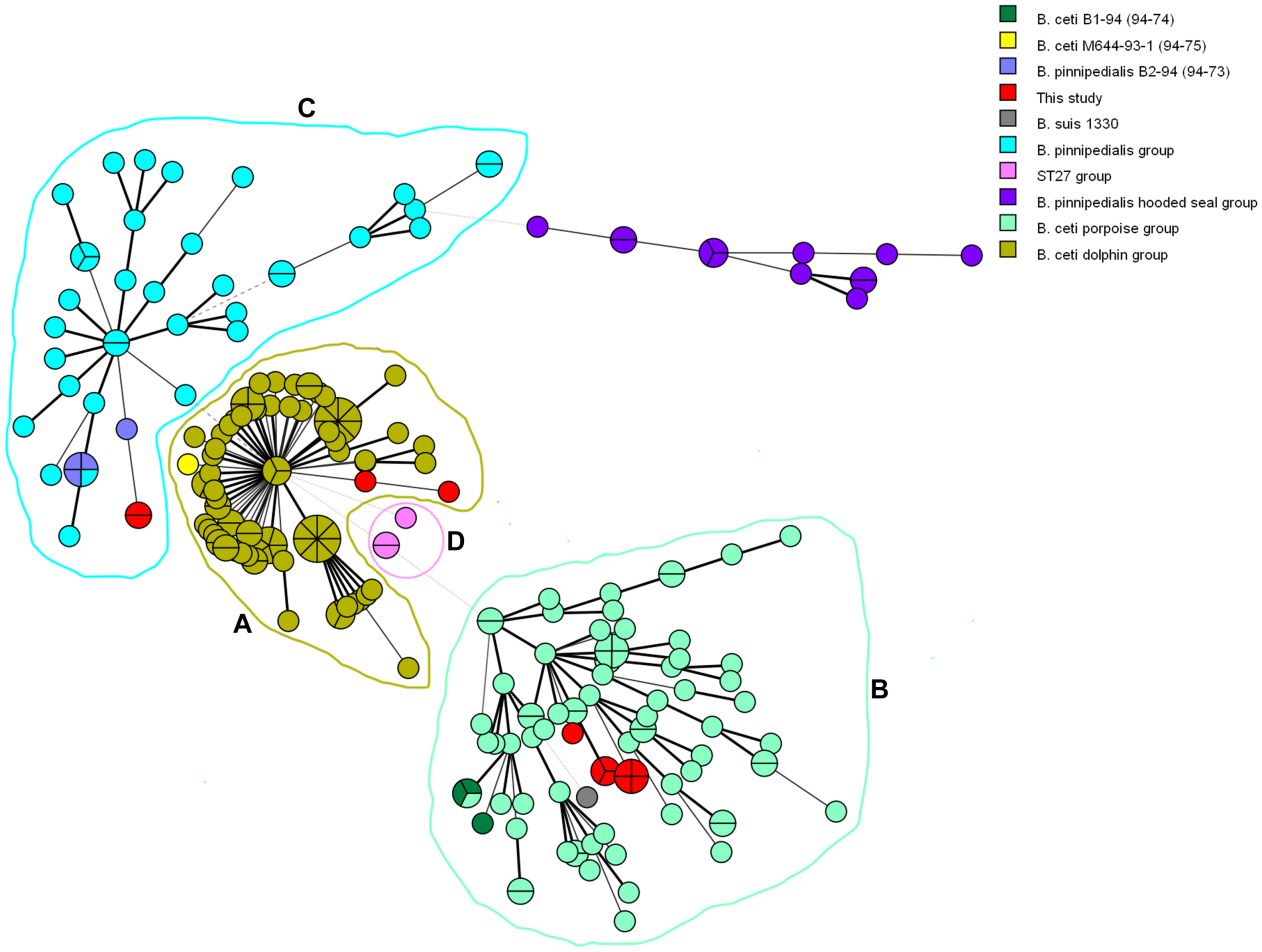
Minimum spanning tree generated from 237 marine *Brucella* MLVA-16 genotypes. Each circle corresponds to an isolate and the size of the circle is proportional to the number of isolates. Strains introduced in this study are colored in red. Analyzed *Brucella* sp. are color-coded, while rings represent clusters **(A–D)**.

Phylogenetic analysis of 237 marine isolates (Garofolo et al., 2013; Isidoro-Ayza et al., 2014; Maquart et al., 2009; Suárez-Esquivel et al., 2017), including additional publicly available sequences from marine and reference strains and the 14 isolates investigated here, is presented in Figure 2. The MLVA-16 results allowed dividing the investigated marine *Brucella* isolates into three clades according to their preferred host: *B. ceti* dolphin type (pattern A, as *B. ceti* M644-93-1), *B. ceti* porpoise type (pattern B as *B. ceti* B1-94), *B. pinnipedialis* seal strains (pattern C, as *B. pinnipedialis* B2-94), and *B. ceti* ST 27 group (pattern D as *B. ceti* 02/611) (Figure 2; Table 3).

**Figure 3:**
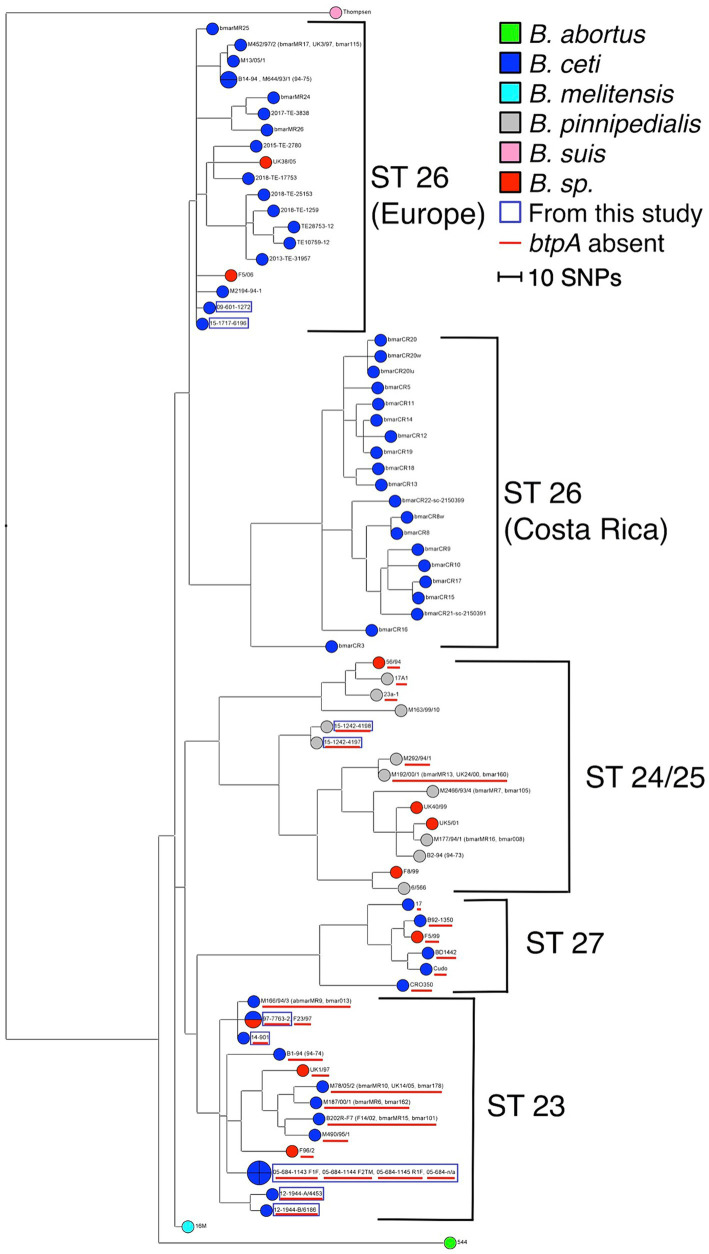
Maximum parsimony analysis of WGS data. The tree has been generated from 7,745 SNPs and rooted with *B. melitensis* 16 M reference genome. The homoplasy index has a value of 1.6% (Parsimony value: 6080). The branch length is proportional to the number of SNPs identified (the scale bar represents the difference of 10 SNPs). Analyzed *Brucella* sp. are color-coded, and the strains issued from this study are marked with a blue square. The absence of the *btpA* gene is marked with a red underscore.

Similarly, WGS analyses clustered marine *Brucella* strains into five clusters: ST 26 (Europe) correspond to *B. ceti* M644-93-1*-*like isolates (pattern A in MLVA), ST 23 to *B. ceti* B1-94-like isolates (pattern B in MLVA), ST 24/25 to *B. pinnipedialis* B2-94-like isolates (pattern C in MLVA), ST 27 to *B. ceti* Cudo-like isolates (pattern D in MLVA), and ST 26 (Costa Rica) (Figure 3).

### 3.4 *In silico* analysis exhibited the absence of specific plasmid and AMR genes, but showed that the btpA virulence gene is variably distributed in different marine *Brucella* ST clusters

No plasmids were detected in any investigated strains when screening the assemblies for entries in the PlamidFinder database. Similarly, no antibiotic resistance genes were found screening NCBI and ResFinder databases. Instead, searching through the CARD and MEGARes databases, one (*mrpF*) and six (*mprF*, *bep C*, *D*, *E*, *F,* and *G*) genes involved in AMR mechanisms were found in all examined genomes including the references. The same tools also identified the *tetC* gene involved in tetracycline resistance only in the *B. ceti* M13-05-1 genome.

**Figure 4:**
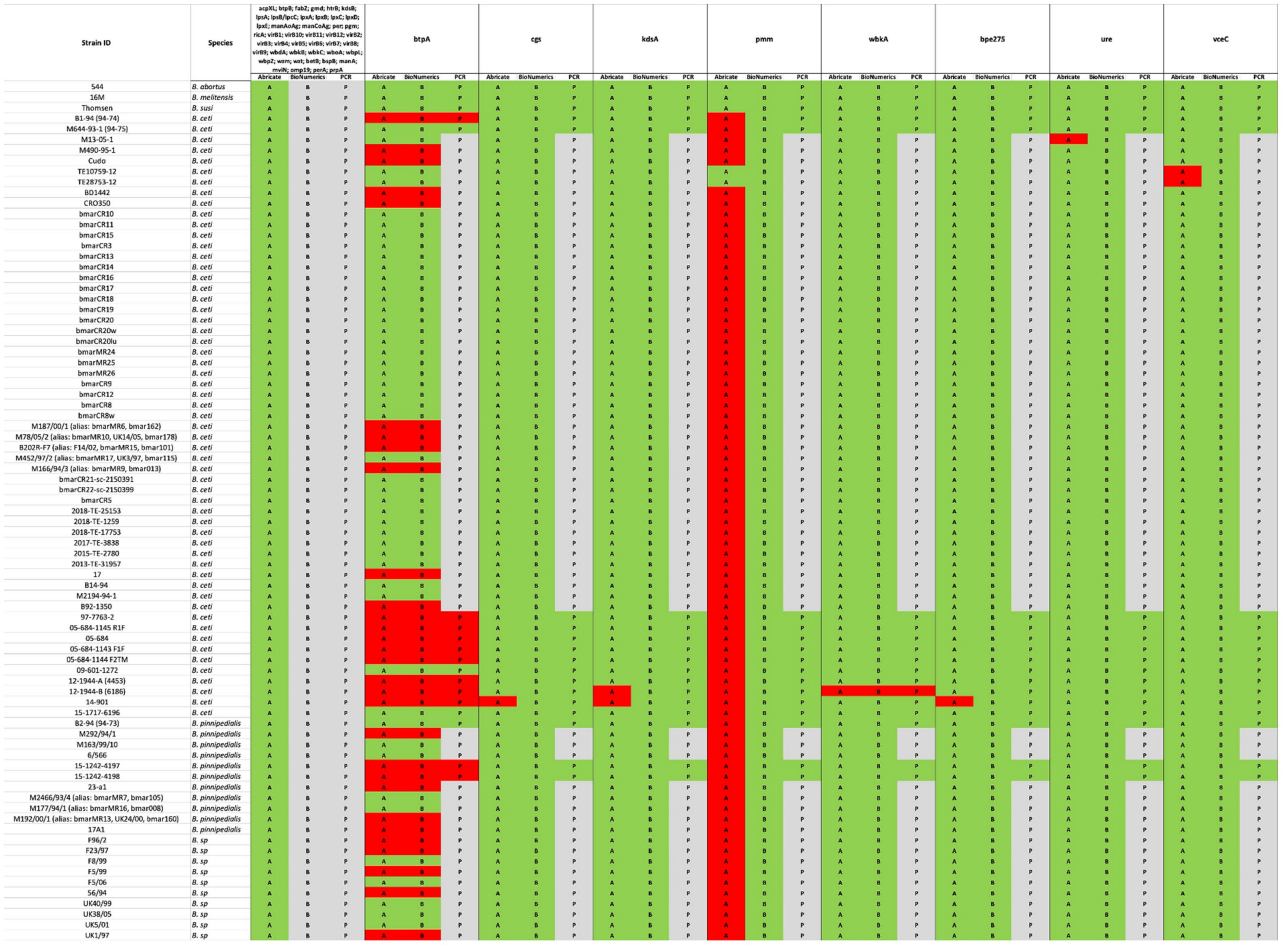
Comparison between *in silico* and experimental molecular analysis for the identification of virulence genes. The *in silico* Abricate (A) and PCR using BioNumerix (B) on available reads were compared to classical PCR (P). The green color signifies the presence, and red absence of the targeted genes, while gray implies that analysis could not be performed.

Exclusively in *B. melitensis*, *B. abortus*, and *B. suis* reference genomes, 53 VGs mainly responsible for host immune evasion, intracellular survival, regulation, and expression of the Type IV secretion system in *Brucellae* were identified, when the VFDB and own BRUgenes databases were examined (Table 4). In *B. pinnipedialis*, *B. ceti* and *Brucella* sp. analyzed genomes, 45 of the 53 VGs were consistently detected, while 8 genes (*btpA*, *cgs, kdsA*, *pmm*, *wbkA*, *bpe275, ure,* and *vceC*) were not found in one or more of the strains screened (Figure 4; Supplementary Table S2). To verify the absence of undetected VGs, an *in silico* PCR using Bionumerics, as well as, a classical PCR with gel visualization (Supplementary Figure S1) was conducted for the available strains. If a gene is detected by *in silico* and/or classical PCR, its presence is considered confirmed (Figure 4; Supplementary Table S2). Moreover, these genes in truncated or pseudo forms were also missing from examined strains.

**Table 4:** Associated virulence and pathogenicity factors detected in *B. ceti*, *B. pinnipedialis,* and *B.* sp. analyzed genomes.

Virulence and Pathogenicity Factors categories	Related genes detected in virulence factor database (VFDB) database	Related genes detected in own BRUgenes database (Rabinowitz et al., 2021)
LPS (lipopolysaccharide), pathogenicity factors, entry, intracellular survival, and immunomodulatory	*acpXL*, *fabZ*, *gmd*, *htrB*, *kdsA*, *kdsB*, *lpsA*, *lpsB*, *lpcC*, *lpxA*, *lpxB*, *lpxC*, *lpxD*, *lpxE*, *manAoAg*, *manCoAg*, *per*, *pgm*, *pmm*, *wbdA*, *wbkA*, *wbkB*, *wbkC*, *wboA*, *wbpL*, *wbpZ*, *wzm*, *wzt*	*manA*, *perA*
Peptidoglycan		*mvinN*
Type IV secretion system and secretion effector proteins	*virB1*, *virB2*, *virB3*, *virB4*, *virB5*, *virB6*, *virB7*, *virB8*, *virB9*, *virB10*, *virB11*, *virB12*	*bpe275*, *bspB*, *vceC*
TIR domain-containing protein immune evasion	*btpA, btpB*	
Rab2 interacting conserved protein A intracellular survival	*ricA*	
CβG (cyclic β-1,2-glucan) intracellular survival	*cgs*	
Biosynthetic of glycine betaine		*betB*
Outer membrane protein		*omp19*
Urease		*ure*
Proline racemases		*prpA*

## 4 Discussion

Marine mammals, at the top of the food chain, are good bio-indicators of the ocean water quality and anthropogenic pressures. Reduction of available food areas pressures marine mammals into smaller or usually inhabitable areas for their species in search of food, increasing close contacts, unusual for cetaceans, which facilitates the spread of infectious diseases, including brucellosis.

Both phenotypic and molecular approaches currently applied to correctly identify the marine *Brucella* species require time, finances, and/or expertise in bacteriological feature interpretation, as well as in genomic analyses, which are cumbersome for field laboratories. Therefore, in this study, we compared the performances of the described molecular tools to correctly classify 12 new isolates from marine wildlife. Furthermore, we used the *in silico* methods to compare these strains to genomes of available marine *Brucella* and evaluate their antimicrobial susceptibility, presence of plasmids, and VGs.

The prevalence of brucellosis among endangered species might impact population dynamics, which requires the adaptation of approaches for species conservation. Bacteriological evidence for discriminating between *B. ceti* and *B. pinnipedialis* is small and depends essentially on phenotypical features common to most isolates reported to date. Classification is thus, mainly directed by host preference. *Brucella* isolates from marine mammals do not produce H_₂_S. Most strains only agglutinate with monospecific anti-A serum, are lysed by Tbilisi, Weybridge, and Izatnagar phages, and can grow in the presence of basic fuchsine and thionine (World Organisation for Animal Health, 2022). Strains infecting pinnipeds need carbon dioxide and are unable to metabolize D-galactose, unlike cetacean strains (Jahans et al., 1997; Banai and Corbel, 2010; Foster et al., 2007; Guzmán-Verri et al., 2012; Jacques et al., 2007). Current spectral databases for protein pattern identification using MALDI-TOF approaches are not sufficient to identify among *Brucella* species, nor separate *Ochrobactrum* sp., yet alone between *B. ceti* from *B. pinnipedialis* (Karger et al., 2013; Mesureur et al., 2018). Thus, identification at the species level of marine isolates requires specific bacteriological and/or molecular tools.

Marine *Brucella* have specific genomic signatures that differentiate them from species affecting mainly terrestrial animals (Cloeckaert et al., 2000). A toolbox of molecular techniques for the identification of marine *Brucella* sp., predominantly based on the study of the polymorphism of the *omp2* gene and the IS*711* fingerprint, is available. The insertion sequence, IS*711,* is present in a greater number of copies in marine *Brucella* (>20 copies) than in terrestrial *Brucella* species, except in *B. ovis* with more than 35 copies (Foster et al., 2007; Ocampo-Sosa and García-Lobo, 2008). Interestingly, a downstream copy of the *bp26* gene (*omp28*) coding for the BP26 periplasmic protein is specific only to marine *Brucella* (Cloeckaert et al., 2000). In addition, similarly to terrestrial *Brucella* that harbors one unique copy of *omp2a* and *omp2b* genes, (except for *B. ovis* – two *omp2a* copies, and the absence of *omp2b*) (Banai and Corbel, 2010), one copy of each gene is reported in pinniped *Brucella*. However, cetacean species show the absence of *omp2a*, but the presence of two *omp2b* copies (Cloeckaert et al., 2001). Therefore, RFLP analysis of the *omp2a*, *omp2b,* and *omp31* genes allows discrimination between *Brucellae* infecting marine mammals (Dawson et al., 2008). The original publication (Cloeckaert et al., 2001) shows three and five distinct restriction patterns of *omp2a* and *omp2b* amplified genes, respectively, for the classification of marine *Brucella*. M(J) pattern, comprising *B. ceti* reference strain B1-94 consists of 79% of harbor porpoise isolates, 15% of dolphins, 4% of seals, and 2% of whale isolates. N(K) pattern, comprising *B. ceti* reference strain M644-93-1, involves 100% isolates from dolphins. L(I) pattern, including *B. pinnipedialis* reference strain M644-93-1, is composed of 95% seal isolates. O(I) pattern comprises of 85% seal isolates. P(I) and Q(I) patterns are reported for isolates from hooded seals and one from Bottlenose dolphin in California (Cloeckaert et al., 2001; Dawson et al., 2008). Using the HRM-PCR SNP-specific marker, described previously by Girault et al. (2022), it was possible to differentiate marine from classic *Brucella* sp. Furthermore, specific SNP for *B. pinnipedialis* can identify this marine species, while *B. ceti* 1 and *B. ceti* 2 SNPs can distinguish between two clades of *B. ceti*. Therefore, HRM-PCR is currently the only easy-to-apply, rapid, and cost-effective molecular method to distinguish marine *Brucella* species, providing high concentration and DNA quality as well as the availability of reference strains.

According to host preference and ocean distribution (Nymo et al., 2011; Suárez-Esquivel et al., 2017), molecular studies confirmed the existence of two distinct groups among cetaceans and pinnipeds and the probable existence of biovars (Moreno et al., 2002) within each marine *Brucella* species. Thus, multi-locus sequencing studies suggested that marine *Brucella* strains are clustered in five sequence types (STs), labeled ST 23 to ST 27. ST 23, ST 26, and ST 27 were associated with infection in cetaceans (porpoises, dolphins, and bottlenose dolphins, respectively), while ST 24/25 was linked with infections in seals (Whatmore et al., 2007). According to VNTR assays, there is a doubt that taxonomy does not reflect the phylogeny of two marine species (Whatmore et al., 2016). Among *B. ceti* isolates, further MLSA, MLVA, and RFLP studies underlined three different groups according to host preference, phenotypic and genomic features: *B. ceti* dolphin type, *B. ceti* porpoise type, and *B. ceti* human type. *B. ceti* porpoise type is more closely related to *B. ceti* human isolates and *B. pinnipedialis* group (Guzmán-Verri et al., 2012). Similarly, *B. pinnipedialis* isolates clustered into two distinct groups, one specific to isolates from hooded seals (found only in central and western North Atlantic), and one clustering other pinnipeds, with respective STs, ST 24/25 (Groussaud et al., 2007). Our MLVA analysis allowed us to organize *B. ceti* into four clades according to their preferred host: *B. ceti* dolphin type (pattern A), *B. ceti* porpoise type (pattern B), and human *B. ceti* (pattern D), separating additional clade from previously published by Guzmán-Verri et al. (2012). MLSAs, i.e., MLVA and MLSA showed a genetic proximity between isolates from marine *Brucella* and *B. suis* (El-Sayed and Awad, 2018; Le Flèche et al., 2006; Whatmore et al., 2006). However, this is not observed with whole-genome SNP analysis, as marine *Brucella* are not directly related to *B. suis* (Sankarasubramanian et al., 2017; Wattam et al., 2014).

Therefore, we decided to use specific molecular approaches designed for *B. suis* to discriminate within marine *Brucella*. RFLP restriction patterns of *omp*2a and *omp*2b genes produced an overall pattern classification as previously described (Cloeckaert et al., 2001). In Suis-Ladder, three patterns were observed, with a complete correlation with MLVA. Our MLVA-16 results are in accordance with the previously reported genomic structures into five clusters: cluster A (ST 26) is exclusively composed of dolphin isolates, cluster B (ST 23) is formed of isolates from porpoises and dolphins, cluster C (ST 24/25) consists of pinniped isolates, including a subcluster C3 for hooded seal strains, and cluster D (ST 27) is represented by only known zoonotic isolates from humans. Finally, deeper WGS analysis confirmed the segregation into four groups for the marine *Brucella* isolates, in total correlation with MLVA and Suis-Ladder results. Additionally, WGS analysis separated the ST 26 group into two sub-clusters based on the geographic origin (European and Costa Rican lineages).

Furthermore, analysis of marine mammalian *Brucella* (*B. ceti*, *B. pinnipedialis,* and *B.* sp.) genomes examined in the CARD and MEGARes databases highlighted the presence in all strains of six genes potentially involved in AMR: the multiple peptide resistance factors *mprF* and the outer membrane efflux proteins *bep C*, *D*, *E*, *F,* and *G*. The overall presence of the MprF factor in highly pathogenic *B. melitensis* and *B. abortus* strains isolated in Egypt and Iran has already been highlighted (Dadar et al., 2023a; Khan et al., 2021), as well in the pan-genome of *B. ceti* and *B. pinnipedialis* (Orsini et al., 2022). MprF plays a key role in the virulence of *Staphylococcus aureus;* among all, it is implicated in resistance to cationic antimicrobial peptides such as gentamycin, moenomycin, and vancomycin (Nishi et al., 2004). Identification of this protein in marine *Brucella* seems to indicate its involvement in the intracellular survival and repulsion of cationic antimicrobials, although Dadar et al. demonstrated no resistance to gentamicin by disk diffusion assays on 40 tested strains (Dadar et al., 2023a). The unique gene that potentially confirms AMR capacity to tetracyclines is *tetC*, detected only in the *B. ceti* M13-05-1 genome using NCBI, ResFinder, CARD, and MEGARes databases. Tetracyclines (including tetracycline and doxycycline) are commonly used for the treatment of brucellosis, and the presence of resistance will have important public health implications. Although specific tetracycline resistance data are not available for marine *Brucella* species, Orsini et al. (2022) speculated that the presence of *tetC* in only two *B. ceti* analyzed genomes (including the M13-05-1 strain) suggests substantial pan-susceptibility to antibiotics of this species. On the other hand, a recent review highlighted how the prevalence of tetracycline and doxycycline resistance in classical *Brucella* species (*B. melitensis* and *B. abortus*) was relatively low (1,7%) but increased over time (Rezaei Shahrabi et al., 2023). To the authors’ best knowledge, currently, no published data are showing the tetracycline resistance test of the mentioned *B. ceti* M13-05-1 strain. Our results are in line with the few studies showing that genes conferring resistance to critically important antibiotics are rare or absent in marine *Brucella*.

In this study, we also analyzed the presence of VG in the genomes of *Brucella* species that mainly infect marine mammals (*B. ceti* and *B. pinnipedialis*), by questioning VFBD and BRUgenes databases, which revealed the presence of an extended set of genes, including those involved in adhesion, invasion, survival within the host cells, and modulation of the immune response (Table 4). The occurrence of the 53 VGs was found only in the genomes of classical *Brucella* species, which is in agreement with data reported in the literature (Dadar et al., 2023a; Khan et al., 2021; Brangsch et al., 2023; Rabinowitz et al., 2021). In contrast to the classical *Brucella* sp. in the genomes of the marines isolates the lack of some genes was evident. Although eight genes were not detected by Abricate search, only the absence of two genes (*btpA* and *wbkA*) was confirmed in one or more genomes, through careful validation by *in silico* and/or classical PCR. Using the same Abricate analysis, the *pmm* gene was not present in any of the marine strains apart from *B. ceti* TE10759-12 and TE28753-12 genomes, confirming the previously published findings (Orsini et al., 2022). However, upon performing the *in silico* and/or classical PCRs, the presence of this gene was confirmed in all marine *Brucella* sp. genomes analyzed (Figure 4; Supplementary Table S2). Misidentification of the *pmm* gene using the VFBD database could be due to the general quality of *de novo* assemblies with the fragmentation of the *pmm* gene through multiple contigs, not allowing proper alignment. This highlights the importance of confirming the results obtained by *in silico* analysis with classical molecular tools, to avoid any possible misinterpretation. The use of complementary tools, such as BioNumerics, allows direct screening of the raw reads, which improves the accuracy of gene detection.

Out of 12 new marine *Brucella* strains described in this study, 2 were found to have rough LPS. The absence of *wbkA* was confirmed in the *B. ceti* 12-1944-B (6186) isolate, described in this study. This gene encodes for the mannosyltransferase, involved in the synthesis of the homopolymeric linear chain of N-formyl-perosamine residues, which are linked via α-1,2 and/or α-1,3-glycosidic bonds into O-polysaccharide (O-PS), a key component of *Brucella* lipopolysaccharide (LPS), the presence of which is responsible for the smooth (S) phenotype (Moriyón and López-Goñi, 1998). Its absence and/or mutation is responsible for the formation of rough (R) *Brucella* (González et al., 2008). This perfectly matches with the phenotype of *B. ceti* 12-1944-B (6186) isolate (Table 1). In contrast, in the other *B. ceti* 15–1717-6196 isolate exhibiting R phenotype, no absence of genes, questioning the VFBD database involved in LPS synthesis was found. O-PS formation depends on a plethora of genes, and the R form can be due to either the absence/silencing of one or more of these genes and/or a mutation in one of them.

Our findings are also particularly intriguing, shedding light on the distinct occurrence of the *btpA* gene, which encodes a Toll/interleukin-1 receptor (TIR) domain-containing protein capable of regulating dendritic cell activation during *B. abortus* infection (Salcedo et al., 2008). Notably, *Brucella*’s TIR-containing proteins, BtpA and BtpB, work in concert to modulate host inflammatory responses during infection by inhibiting dendritic cell activation (Salcedo et al., 2013) and influence cellular energy metabolism by hydrolyzing NAD^+^ (Coronas-Serna et al., 2020). While the precise targets of TIR-containing *Brucella* effector proteins remain to be elucidated, significant differences emerge between these two proteins. For example, BtpA has a known role in specifically affecting macrophage TNF-α secretion (Salcedo et al., 2013). In contrast to the pan-genome analysis of *B. ceti* and *B. pinnipedialis* genomes, which suggests a consistent presence of both proteins (Orsini et al., 2022), our data reveal the absence of BtpA within *B. ceti* ST 27 and 23 clusters (Figure 3), which was also confirmed by classical PCR analysis. Furthermore, its distribution within the genomes of *B. pinnipedialis* (ST 24/25 cluster) shows a division into two subclades, with some maintaining its presence and others lacking it.

In conclusion, the HRM-PCR or Suis-Ladder multiplex PCR can be easily set up by any laboratory familiar with PCR methods, and the ability to reliably identify marine mammal brucellae has been demonstrated in this study. In terms of costs, both methods are cost-effective approaches for any laboratory. Within a One-Health worldwide context, the ability to easily characterize a *Brucella* strain isolated from a marine mammal is a powerful tool for every lab in the world. Furthermore, the *in silico* analyses should be verified by molecular approaches, to better classify marine *Brucella* strains regarding the presence of AMR and VGs.

## Data Availability

The datasets presented in this study can be found in online repositories. The names of the repository/repositories and accession number(s) can be found in the article/Supplementary material.

## Supplementary material

Supplementary Figure S1 Gel visualization of missed virulence genes.

Supplementary Figure S2 Original gel visualization of Suis-Ladder patterns of B. suis biovars, B. canis and marine Brucella reference strains.

Supplementary Table S1 List of genomes sequences and DNA available in laboratory used in this study.

Supplementary Table S2 All data concerning detection of 53 virulence genes with in silico methods (Abricate=A and BioNumeriscs=B) and gel visualization of PCR (PCR=P).
