# Supplementary figures and images for: Combination of in silico and molecular techniques for discrimination and virulence characterization of marine Brucella ceti and Brucella pinnipedialis
Source: Front Microbiol. 2024 Sep 18;15:1437408. doi: 10.3389/fmicb.2024.1437408 (PMC11444999; doi:10.3389/fmicb.2024.1437408)

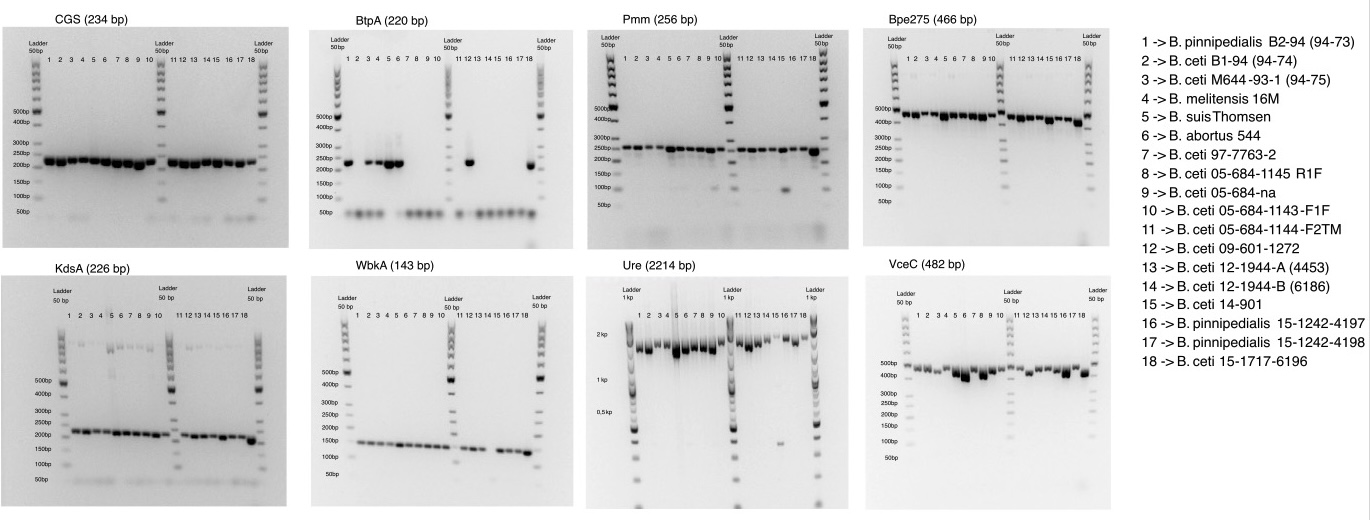

Supplement: Supplementary Figure S1 — Gel visualization of missed virulence genes. [file Image_1.JPEG]

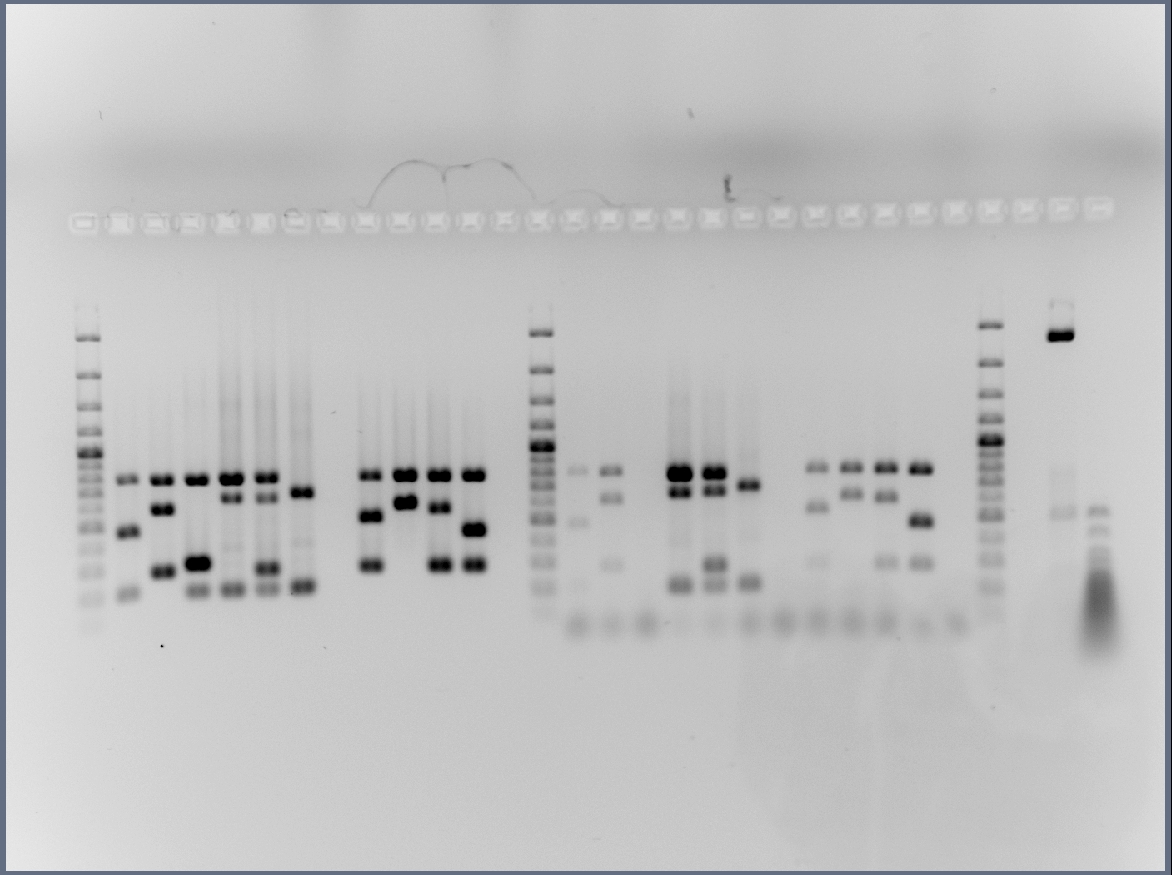

Supplement: Supplementary Figure S2 — Original gel visualization of Suis-Ladder patterns of B. suis biovars, B. canis and marine Brucella reference strains. [file Image_2.JPEG]
